# Genomic Selection in Commercial Perennial Crops: Applicability and Improvement in Oil Palm (*Elaeis guineensis* Jacq.)

**DOI:** 10.1038/s41598-017-02602-6

**Published:** 2017-06-06

**Authors:** Qi Bin Kwong, Ai Ling Ong, Chee Keng Teh, Fook Tim Chew, Martti Tammi, Sean Mayes, Harikrishna Kulaveerasingam, Suat Hui Yeoh, Jennifer Ann Harikrishna, David Ross Appleton

**Affiliations:** 1 Biotechnology & Breeding Department, Sime Darby Plantation R&D Centre, Selangor, 43400 Malaysia; 2 Department of Biological Sciences, National University of Singapore, Singapore, 117543 Singapore; 3 School of Biosciences, University of Nottingham, Sutton Bonington Campus, Loughborough, LE12 5RD UK; 4 Institute of Biological Sciences, University of Malaya, 50603 Kuala Lumpur, Malaysia; 5 Centre of Research in Biotechnology for Agriculture (CEBAR), University of Malaya, 50603 Kuala Lumpur, Malaysia

## Abstract

Genomic selection (GS) uses genome-wide markers to select individuals with the desired overall combination of breeding traits. A total of 1,218 individuals from a commercial population of *Ulu Remis* x *AVROS* (UR x AVROS) were genotyped using the OP200K array. The traits of interest included: shell-to-fruit ratio (S/F, %), mesocarp-to-fruit ratio (M/F, %), kernel-to-fruit ratio (K/F, %), fruit per bunch (F/B, %), oil per bunch (O/B, %) and oil per palm (O/P, kg/palm/year). Genomic heritabilities of these traits were estimated to be in the range of 0.40 to 0.80. GS methods assessed were RR-BLUP, Bayes A (BA), Cπ (BC), Lasso (BL) and Ridge Regression (BRR). All methods resulted in almost equal prediction accuracy. The accuracy achieved ranged from 0.40 to 0.70, correlating with the heritability of traits. By selecting the most important markers, RR-BLUP B has the potential to outperform other methods. The marker density for certain traits can be further reduced based on the linkage disequilibrium (LD). Together with *in silico* breeding, GS is now being used in oil palm breeding programs to hasten parental palm selection.

## Introduction

Genomic Selection (GS) is defined as marker assisted selection using markers representing all QTL in the genome^1^. These markers are used to build a predictive model using individuals with known genotypic and phenotypic information. With this model, genomic estimated breeding values (GEBVs) for the desired trait can be calculated and used to rank the individuals with unknown phenotype for subsequent selection. This method was initially developed for the use in cattle breeding, and is revolutionizing the industry^2^. Since then, this method has been introduced in plant breeding, inclusive of wheat, maize^3^ and pines^4^. However, in most crops, GS approaches are still in the research phase and yet to be applied in a large scale breeding program^5^. With the high marker density required for GEBV estimation, genotyping cost increases and the economic viability of GS remains in question for certain applications. In commercial perennial crops breeding, such as oil palm, each generation or selection cycle involves multiple crosses which easily produce far larger progeny numbers than in cattle breeding. A reduction in marker density will lower the genotyping cost. Thus, in order to maximize the value of GS in commercial crops such as oil palm, marker density needs to be optimized.

Being the major oil crop of the world, oil palm (*Elaeis guineensis* Jacq.) accounted for 35% of the world’s vegetable oil consumption in 2015, with a steady upward trend seen since the late 90’s^6^. However, the realized oil yield in Malaysia remains stagnant at the range between 3.0 to 4.0 t/ha/yr, for more than 25 years^7^. Breeding gain needs to be increased significantly to address new challenges such as land degradation, climate change and agricultural land constraints. To provide a solution while ensuring a sustainable future, marker assisted breeding/selection has been introduced into oil palm breeding programs. The implementation of these programs requires genetic markers, which are more readily discovered now through resequencing, after the reference genome of oil palm became available^8^.

Examples of markers acquired recently are the *SHELL* markers, which are capable of distinguishing fruit forms in oil palm: *dura*, *tenera* and *pisifera* ^9^. The *dura* fruit form is known to have a thick shell and absence of fiber ring, *tenera* has a thin-shell with a fiber ring, while *pisifera* is shell-less with a fiber ring and is often female sterile. With shell thickness being inversely correlated with mesocarp thickness in the oil palm fruitlet, *tenera*, the hybrid of *dura* and *pisifera*, is preferred and exploited commercially. Characterization of simple Mendelian traits such as fruit form requires only a few markers. Most of the traits which are economically important, however, are quantitative and complex in nature and require whole genome coverage of markers. Through the calculation of GEBV, GS provides a potential solution to select for these traits in breeding populations.

GS is a relatively new approach in oil palm breeding that enable early selection of elite materials, maximizing genetic gains over generations. The principle of GS has been described in oil palm through simulated data^10^ and the implementation of it using SSR has been proven to be feasible^11^. Still, to implement GS in oil palm requires more in depth studies regarding the use of such high density data in the characterization and representation of the genetic component of the traits of interest. For this purpose, a large commercial *tenera* population was selected to assess GS applicability in terms of selection response of the targeted traits and prediction accuracy. With genotyping the entire genome being expensive, the cost of GS implementation is generally high. A method that reduces markers without compromising on the prediction accuracy will definitely make GS more economically viable in all plant breeding programs, inclusive of oil palm.

## Results

### Kinship Coefficient Estimate

The kinship coefficient of the assayed UR x AVROS population averaged 0.23, ranging from 0.00 to 0.74, with > 74% of the individuals having relatedness between 0.20 and 0.40. Kinship coefficients distribution of this population can be found in Supplementary Fig. 1.

### Genomic Heritability

**Figure 1:**
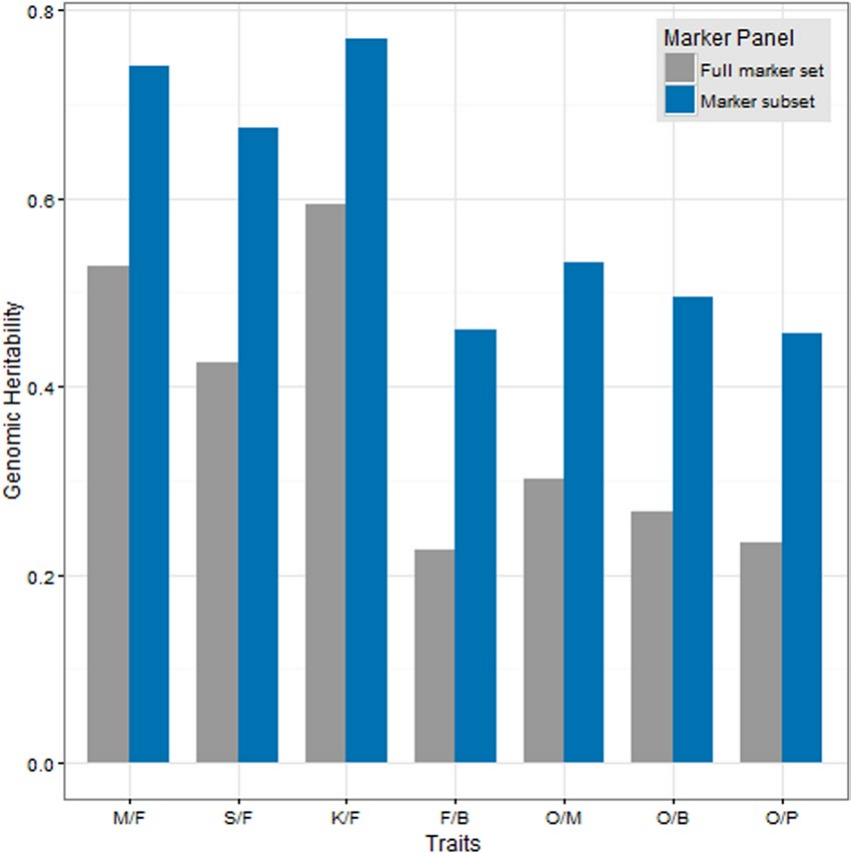
Genomic heritability estimate for 7 traits using the full marker set against marker subset based on association score.

Based on an association score cut-off of 1.3, approximately 3,500 markers were used for the heritability estimation of S/F and 3,900 were used for M/F, K/F, F/B, O/M, O/B, O/P traits (Fig. 1). The traits, ranked based on heritability from the highest to the lowest were K/F, M/F, S/F, O/M, O/B, F/B and O/P. Overall, genomic heritability estimated using the full marker set and the marker subset showed good correlation (*r* = 0.99) (Supplementary Fig. 2), with higher heritability being estimated using the subset. M/F, S/F and K/F were classified as traits with high heritability, whereas F/B, O/B and O/P were classified as traits with medium heritability.

### Genomic Selection

**Figure 2:**
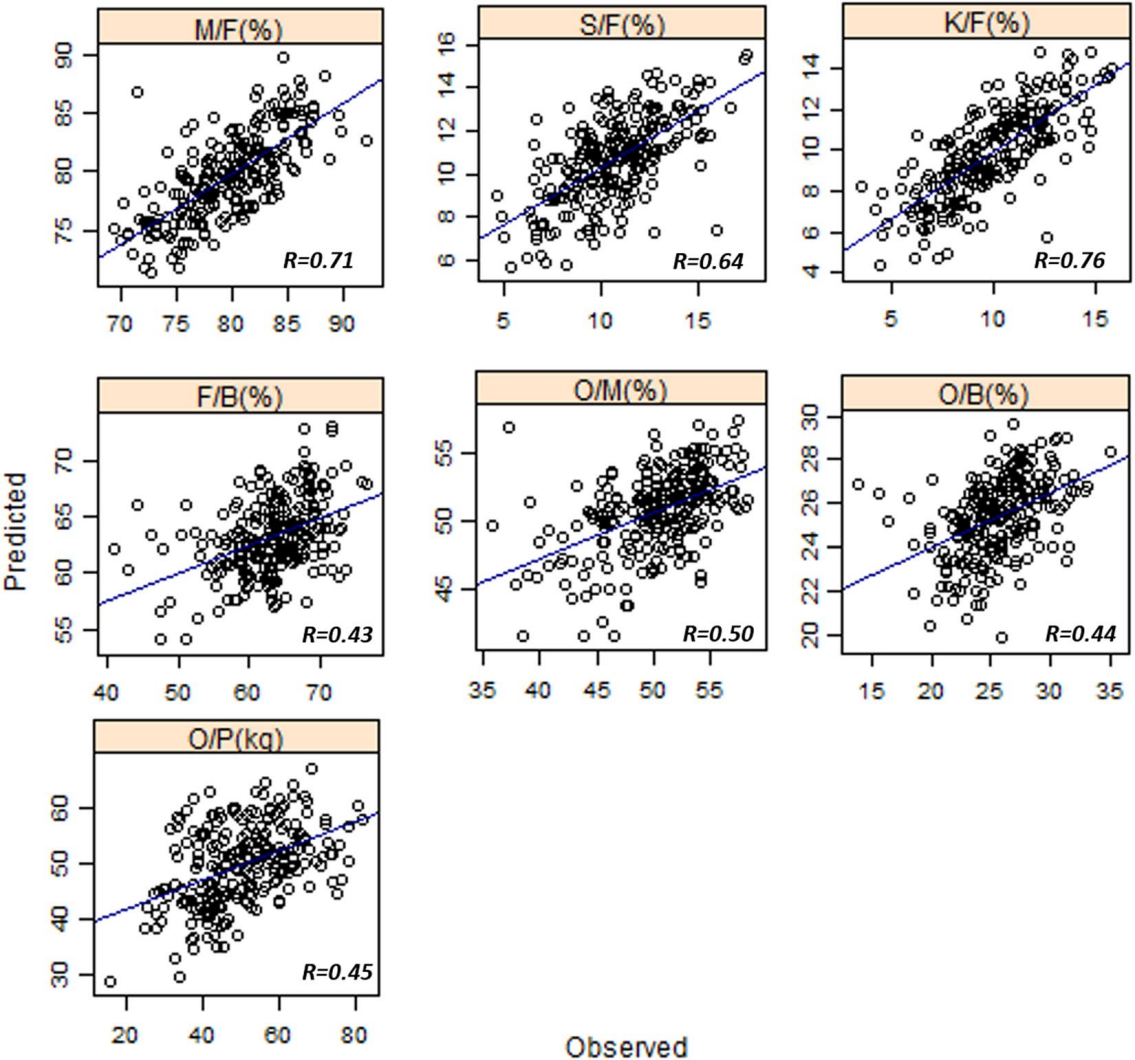
Representative plots for predicted trait values versus the observed trait values for all traits.

Overall, the prediction accuracy ranged from 0.43 to 0.75 after averaging them by trait across all methods (Table 1). The trait with the highest accuracy was K/F (0.75) and the ones with lowest accuracy were O/B and F/B (0.43). RR-BLUP and all Bayesian methods performed almost equally in predicting the traits. Representative plots that show the relationship between predicted trait values versus observed trait values were shown in Fig. 2. Given these accuracy values, the estimated selection response for all the traits were as reported in Supplementary Table 1.

**Table 1:** Prediction accuracy (with standard deviation in brackets) for 7 traits based on RR-BLUP and Bayesian methods.

	M/F	S/F	K/F	F/B	O/M	O/B	O/P	Average by Method
RRBLUP	0.71 (0.02)	0.63 (0.02)	0.75 (0.02)	0.44 (0.05)	0.50 (0.03)	0.43 (0.06)	0.47 (0.07)	0.56
BA	0.72 (0.02)	0.67 (0.02)	0.75 (0.02)	0.43 (0.05)	0.50 (0.03)	0.43 (0.06)	0.46 (0.07)	0.57
BC	0.71 (0.02)	0.63 (0.04)	0.75 (0.02)	0.44 (0.05)	0.50 (0.03)	0.43 (0.06)	0.47 (0.07)	0.56
BRR	0.71 (0.02)	0.63 (0.02)	0.75 (0.02)	0.44 (0.05)	0.50 (0.03)	0.43 (0.06)	0.47 (0.07)	0.56
BL	0.69 (0.03)	0.63 (0.03)	0.74 (0.03)	0.42 (0.04)	0.50 (0.03)	0.42 (0.06)	0.43 (0.07)	0.55
Average by Trait	0.71	0.64	0.75	0.43	0.50	0.43	0.46

For both traits with high and medium heritability, Bayesian methods and RR-BLUP performed almost equally. Within the traits with high heritability, the average accuracy for M/F was 0.71 (RRBLUP 0.71, BA 0.72, BC 0.71, BRR 0.71, BL 0.69), S/F was 0.64 (RRBLUP 0.63, BA 0.67, BC 0.63, BRR 0.63, BL 0.63) and K/F was 0.75 (RRBLUP 0.75, BA 0.75, BC 0.75, BRR 0.75, BL 0.74). The prediction accuracy for O/M across all methods was 0.50. For the traits with medium heritability, the average accuracy for F/B was 0.43 (RRBLUP 0.44, BA 0.43, BC 0.44, BRR 0.44 and BL 0.42), O/B was 0.43 (RRBLUP 0.43, BA 0.43, BC 0.43, BRR 0.43 and BL 0.42) and O/P was 0.46 (RRBLUP 0.47, BA 0.46, BC 0.47, BRR 0.47 and BL 0.43).

**Figure 3:**
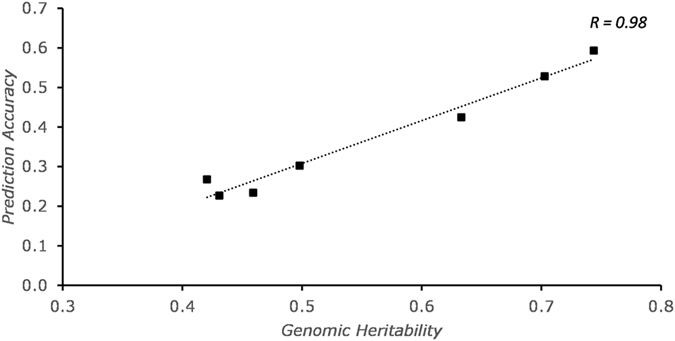
Correlation between prediction accuracy and genomic heritability for all traits.

Figure 3 was generated using marker subset-estimated genomic heritability and prediction accuracies of traits averaged across different methods. A high correlation (*r* = 0.98) between prediction accuracy and genomic heritability was observed, which indicates that the higher the heritability, the higher the accuracy. For subsequent analysis, GS accuracy assessments for both RR-BLUP-B and LD-based marker filter were carried out using RR-BLUP as the basis for comparison.

### Marker Reduction by Association/RR-BLUP-B

**Figure 4:**
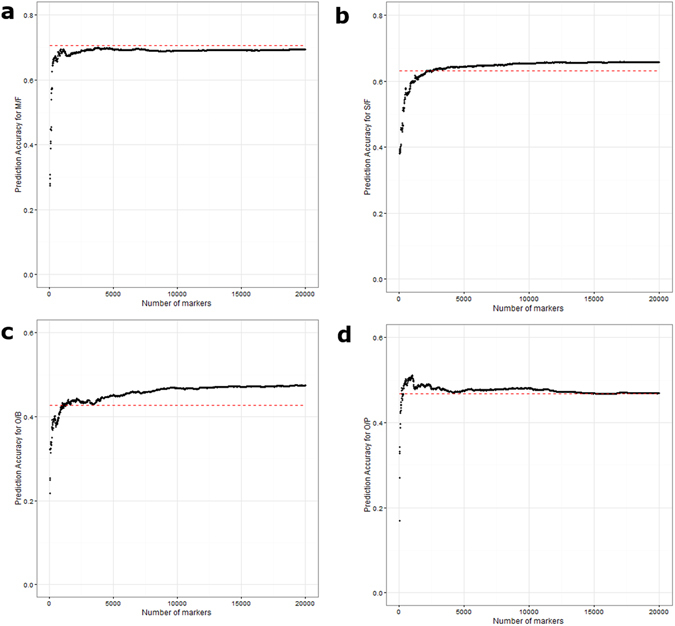
Average prediction accuracy for 4 traits across different marker densities using RRBLUP-B method: (**a**) M/F, (**b**) S/F, (**c**) O/B and (**d**) O/P. (**a**) RRBLUP-B result for M/F with accuracy 0.7 at 3,800 markers (RRBLUP 0.71), (**b**) for S/F with accuracy 0.66 at 10,530 markers (RRBLUP 0.63), (**c**) for O/B with accuracy 0.48 at 12,500 markers (RRBLUP 0.43) and (**d**) for O/P with accuracy 0.51 at 990 markers (RRBLUP 0.47). The red dotted line represents accuracy acquired from RR-BLUP for each trait.

The result for marker reduction differed according to traits. For M/F (Fig. 4a), the highest accuracy peak of 0.7 was achieved with 3,800 markers. This peak was slightly lower than 0.71 achieved from RR-BLUP. A small peak at 0.69 was observed at 840 markers. For S/F (Fig. 4b), the graph was a half sigmoid with the highest accuracy at 0.66, requiring 10,530 markers, surpassing 0.63 from RR-BLUP. For O/B (Fig. 4c), RR-BLUP B outperformed RR-BLUP at 870 markers, before hitting a local peak of 0.44 at 2,150 markers, and plateauing at 12,500 markers with 0.48 accuracy. The maximal accuracy acquired for O/P (Fig. 4d) was 0.51 at 990 markers, before dropping off and plateauing at 0.47, which is the accuracy acquired from RR-BLUP. More information is available in Supplementary Table 2.

**Figure 5:**
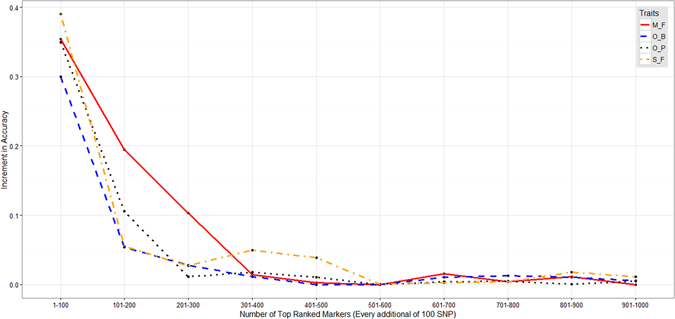
Prediction accuracy increment for every additional 100 SNP for M/F, S/F, O/B and O/P traits.

Based on Fig. 5, the optimal marker density before reaching minimal accuracy increment was 300 SNPs for M/F (accuracy 0.66), 400 SNPs for S/F (accuracy 0.54), 200 SNPs for O/B (accuracy 0.39) and 200 SNP for O/P (accuracy 0.48).

**Figure 6:**
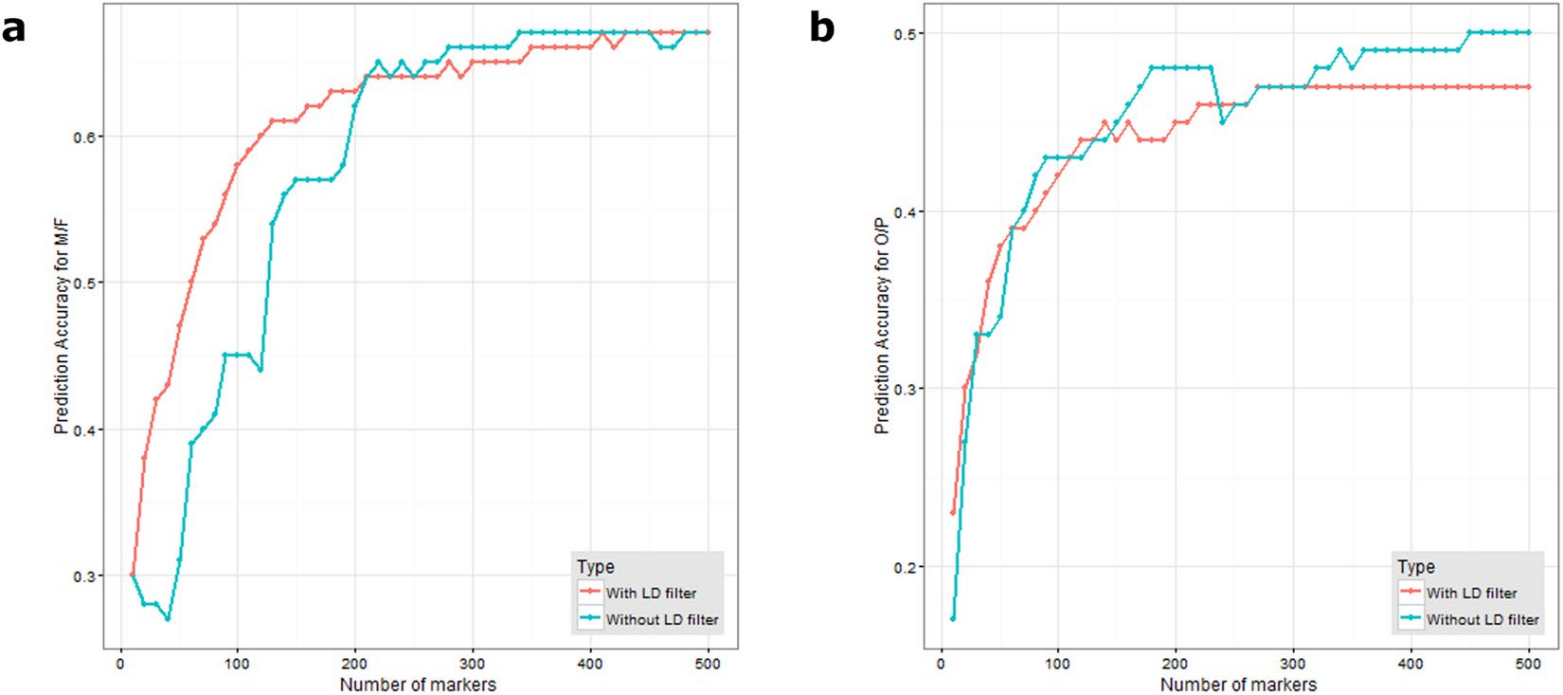
Comparison of prediction accuracy for ascending number of markers, with or without LD filter. (**a**) GS accuracy for RRBLUP-B markers filtered based on LD for M/F. (**b**) for O/P.

### Marker Reduction by LD

Marker reduction by LD was carried out for M/F and O/P using the first 300 ranked markers for each trait. Figure 6a shows that there were “drags” for accuracy of M/F without the LD-filter at 10–40, 60–80, 90–110, 150–190 markers. From the result, M/F was able to achieve an accuracy of 0.60 with 120 markers using the LD-filter, as compared to 200 markers required without the LD-filter. This method did not work for O/P (Fig.﻿ 6b). In fact, in this trait this approach caused a slight decrease in accuracy. More information is available in Supplementary Table 3.

## Discussion

A commercial *tenera* population derived from multiple UR x AVROS families was studied. The average kinship within this population was calculated to be 0.23. This result agreed with known pedigree records, which mainly comprised of half siblings. A few of the individuals were found to be unrelated, which might be due to uncommon ancestors used in the founding population. The UR background is generally chosen in oil palm breeding for high bunch number and a high ratio of female: male inflorescences^12^, with the AVROS pollen parent conferring the properties of growth uniformity, precocity and high mesocarp oil content. This combination results in a hybrid population suitable for commercial oil production, with improved oil yield traits such as high O/M and low S/F. With high oil yield being one of the key characteristics of this population, the traits selected for this study were traits closely related to it. These traits included M/F, S/F, K/F, F/B, O/M, O/B and O/P. The selection response of each trait corresponded to their heritability estimates.

In this study, we found that the heritability estimates correlated well with the conventional heritability of published phenotypes (K/F, M/F, O/B and F/B)^13^. To distinguish this from the conventional heritability, the heritability estimates are referred to as “genomic heritability”. This estimate of genomic heritability is based on whole genome regression, where the regression on the markers is used to explain the phenotypic variance^14, 15^. This differs from conventional heritability calculation, which is usually calculated from the slope of parent-offspring regression^16^. The calculation of genomic heritability is affected by the number of markers used under a linear model. Selection of the whole marker set assumes that every QTL is represented by a marker which accounts for a certain amount of the total trait variance, whereas the subset assumes that only certain QTL are responsible for the majority of trait variance^1^. Since the latter models the true biology better, higher heritability was estimated for all traits using this approach in this study. From another perspective, the use of all the markers in linkage equilibrium with QTL introduces more inconsistencies into the estimates of genomic heritability^15^. The problem with the use of marker subsets, however, is that defining every single QTL controlling a trait is difficult, and setting an association analysis cut-off for complex traits is rather subjective. Although the absolute heritabilities calculated were different, there was an almost perfect correlation between the values calculated from both methods (*r* = *0.99*). Since the role of heritability in GS is to provide an estimate of the response to artificial selection for a trait, the relative comparison of trait heritability within a single method will still provide important information. One method for determining the marker density is to select them based on the highest prediction accuracy achievable under RR-BLUP B. However, this could only be done if every single QTL in the genome is represented by at least one marker. Even so, this method of calculation still slightly underestimates the true heritability, since it assumes that the causal variant for each QTL has been captured, whereas in fact, many of the markers are merely in LD with them^17, 18^. With this, genomic heritability should only be estimated by this method with the basic condition that the marker density is representative of the entire genome, a condition that is also a must in GS.

The earliest GS method, namely RR-BLUP did not detect QTL and assumed that all markers explain the same amount of phenotypic variance^1^. This was followed by the Bayesian methods that allow for different marker variances, which invariably indicate a preference of certain markers over others in modeling for a trait^19, 20^. Both methods have met with varying degrees of success^21, 22^. From our study, the accuracies for all the traits under both methods were generally high, ranging from 0.40 to 0.70. The current accuracy can be further improved with a larger training population. The prediction accuracies achieved were almost in a perfect correlation with the trait genomic heritability, with the highly heritable traits having high accuracy and *vice versa*. Similar results have been reported in other crops^23, 24^. As both genetics and environment play an important role in determining the phenotype, a high heritability indicates a high genetic effect for the trait. Since GS captures the additive genetic effect, this would explain the good correlation between heritability and prediction accuracy. It has been reported that Bayesian methods are better options than RR-BLUP for traits with a few large QTL effects^25^. We observed that there is only a slight advantage in using Bayesian methods as compared to RR-BLUP (M/F, S/F, K/F, O/M). Similarly, in traits controlled by many small QTL effects (F/B, O/B, O/P), there is also only a slight increase in accuracy using RR-BLUP as compared to Bayesian methods. This is probably due to the fact that all important QTL were in LD with at least one polymorphic marker and represented in the genotyping array, and that we have used sufficient iterations for the Bayesian modeling step.

In order for GS to perform well, the LD *r* ^*2*^ ^26^ threshold of more than 0.2 between a marker and a QTL was found to be important^2^. In practice, since the QTL is not known, LD could only be calculated between markers used in the study. LD could be used as a method to reduce the marker density required, since markers in the same LD essentially carry the same information regarding the QTL. This method is particularly useful for traits where the QTL are few and have long LD regions with many markers, such as for M/F. For traits controlled by more QTL, inclusive of O/P, this method might not be effective. A way to distinguish between these two cases is by identifying accuracy drags when the accuracy per marker graph shape is not a perfect half sigmoid. A note of caution when applying this filter is that there might be a slight reduction in the global maximum accuracy. This is because it is difficult to set a common cut-off as every LD has a different length. A case by case approach would be better than setting a global cut-off. Even though the filtering by the LD method provides an option to reduce the number of markers required, in order to maintain prediction accuracy, a method that selects markers best representing the QTL must precede this step. RR-BLUP B provides a solution to this problem.

A modification of RR-BLUP, termed RR-BLUP B, was found to be as effective as the Bayesian method^27^. This method ranks and subsets the SNPs based on marker effect. The accuracy of RR-BLUP B is dependent on the markers selected, which in turn is dependent on the association score cut-off being set. In Fig. 4, we show that RR-BLUP B has the potential to outperform RR-BLUP when the threshold being set for marker selection is optimal for accuracy. This result is similar to that reported in rice^28^. Also, this method required shorter running time. Each of the diagrams in Fig. 4 represents a different potential scenario for RR-BLUP B. For both M/F and S/F, the association analysis detected important QTL, resulting in fewer markers required before hitting the accuracy close to RRBLUP. However, for the case of M/F, the accuracy from RR-BLUP B (0.70) was slightly lower than RRBLUP (0.71). In the case of O/P, the global maximal accuracy was achieved before a subsequent reduction in accuracy with additional markers. This reduction of accuracy trends towards the accuracy of RRBLUP for the same trait. For O/B, a local maximum (0.44) was achieved through the association ranking approach. The global maximum was achieved after 20,000 markers, with accuracy estimated to be 0.48, higher than that of RR-BLUP (0.43). These graph patterns are largely similar to results obtained from loblolly pine^27^. The principle behind RR-BLUP B is to use the most informative/largest effect marker up-front, and is therefore dependent on the efficiency of marker selection. Given the fact that the population in this study was split into smaller sets before attempting association studies, there were inconsistencies in the QTL detection due to the relatively small sample size, and this best explains the result observed for M/F and O/B. With sufficient sample size, the accuracy of all the traits can be improved. Association studies, therefore, are not only useful in detecting large QTL effects, but also offer the possibility of ranking QTL with small effects.

With the associated markers showing more importance than other markers in the RR-BLUP B model, and RR-BLUP-B outperforming RR-BLUP in most cases, it might be more apt to define genomic selection as marker assisted selection that considers all markers representing all QTL responsible for a trait, instead of the entire genome. The problem that remains is what cut-off needs to be set for association or marker effects for the clear definition and representation of a QTL. This decision directly affects the number of markers to be used. On the other hand, the greatest strength of RR-BLUP B is the ability to control for the number of markers to be used in the model. In this study, approximately 200–400 top-ranked SNP markers for each trait (M/F, S/F, O/B and O/P) were required to achieve the optimal prediction accuracy. In other words, only the top 1–2% of the whole-genome SNP markers need to be deployed in GS implementation. The increment in accuracy was minor or negligible when the number of markers exceeded 400, when used with these models. The higher the number of markers, the higher the cost of implementation. Therefore, the number of markers to be selected for GS is also an economical decision instead of a purely scientific decision.

Characterization of simple traits requires only a few markers. Important traits in plants, for example O/P in oil palm, are complex in nature and require markers representative of the whole genome. The implementation of GS consolidates the effects of these markers into a GEBV, enabling ranking of the best performing individuals. The calculation of genomic heritability allows us to know the selection response of the trait of interest, which also implied the GS accuracy. The practical application of GS, however, is dependent on the cost, which is influenced by the number of markers used. Our results with marker sub-setting and LD filtering showed that marker reduction is possible without much reduction in accuracy. While it is recognized that the subsets of effective markers may be different according to the targeted traits, with a crop such as oil palm with large progeny numbers from a long breeding cycle, it would be feasible and practical to minimize genotyping costs by selecting the best markers for each trait and modeling them independently. In this study, our focus has been on selecting the best performing progenies for planting. However, the selection of parents is of utmost importance for commercial plant breeding programs. Therefore, instead of selecting for progenies, the models built can also be used in combination with *in silico* breeding, through crossing simulation, to select the best parents. The GEBV calculated for the simulated progenies can be used as the means to rank and select the best parents. This will reduce the cost even further, making genomic selection an even more attractive option for commercial perennial crop breeding.

## Materials and Methods

### Plant Materials and Data

A total of 1,218 *tenera* palms derived from multiple UR x AVROS crosses^13^ was selected as the study population. The population is maintained at Sime Darby R&D Centre, Malaysia and was phenotyped for the traits of M/F, S/F, K/F, F/B, O/M, O/B and O/P according to the industry standard^29, 30^. The phenotying of these traits, except O/P were conducted under bunch analysis to generate reliable mean values with at least 3 bunches per palm. The O/P trait was then calculated based on the multiplication between 4-year average fresh fruit bunch (FFB) and O/B.

The genomic DNA of each palm was extracted from 100 mg of dried leaf tissue and purified using the DNAeasy Plant Mini Kit (Qiagen, Germany). Genotyping of this population was done using the OP200K SNP array^31^ and 92,057 SNPs were found to be polymorphic and were used for GS purposes.

### Kinship Coefficient Estimate

Kinship coefficients within the population were calculated based on pairwise comparison between all the individuals used in this study. A total of 15,000 SNPs were selected at random from the full SNP genotypes. The resulting file was reformatted using an in-house Perl script. Kinship was estimated using the *related* package^32^ implemented in R. Lynch & Li method^33^ was selected to calculate the pairwise kinship. This was done using the command “coancestry” and setting “lynchli” as 1. The bootstrap replication parameter was set at 100 and the inbreeding was set as False.

### Genomic Heritability

Heritability was calculated based on a linear model using all informative SNP markers^15, 17^. Another calculation of heritability was based on marker subsets with selection criteria as defined under the RR-BLUP B section below. However, in this case, we set the association scores (−log(*P*-value)) cut-off to be 1.3.

### Genomic Selection

The different methods studied were RR-BLUP, Bayes A (BA), Bayes Cπ (BC), Bayes Ridge-Regression (BRR) and Bayes Lasso (BL). RR-BLUP was implemented using the *rrBLUP* package^34^ and all the Bayesian methods were implemented using the *BGLR* package^35^. For the Bayesian methods, the number of iterations was set to 20,000, with the first 2,500 discarded as burn-in. For BL, the additional parameter of lambda was set to 25, type as gamma, rate as 1e-4 and shape as 0.55. A 5-fold cross validation was carried out, where the data was divided into 5 subsets, 4 of which (975 individuals) were used for modeling, and the last subset (243 individuals) was used for validation. This process was repeated 5 times, until all subsets were used for both modeling and validation. Correlation between the GEBV and observed trait value was used as a measurement for prediction accuracy^24, 36, 37^. Selection response for each of the traits was estimated based on the top 25% best performing individual as compared to the overall mean of unselected individuals.

### Marker Reduction by Association/RR-BLUP B

Due to the large number of iterations required, only two traits with high heritability (M/F and S/F) and another two with medium heritability (O/B and O/P) were selected. For each of the traits, a genome-wide association study (GWAS), using the same method as described in our previous publication^38^, was carried out independently for each iteration during the cross validation step. Markers were ranked according to the association score. Subsequently, a stepwise increase by 10 markers, up to 20,000 markers, was carried out for model training. Genomic selection was carried out as described in the previous section for RR-BLUP. The accuracy for each marker set was defined based on the mean accuracy achieved. In addition, we have also looked at the optimal number of markers by calculating the accuracy increment for every 100 markers. The optimal marker density was defined as the point where the increment in the number of markers causes an improvement in accuracy of less than 0.05.

### Marker Reduction by LD

To illustrate LD-based marker reduction, the traits M/F and O/P were selected. LD was estimated using PLINK^39^ on top of association-ranked SNP. Pairwise LD calculation was done for each of the selected markers using R-squared and a cutoff at 0.6. Only one SNP was selected from a LD. Prediction accuracy was determined by RR-BLUP using the parameters described above. To illustrate the result, the prediction accuracy for the first 600 markers selected was plotted for both cases, with and without the LD filter.

## Electronic supplementary material

Supplementary table 2

Supplementary information
