# Supplementary material for: Genomic Selection in Commercial Perennial Crops: Applicability and Improvement in Oil Palm (Elaeis guineensis Jacq.)
Source: Sci Rep. 2017 Jun 6;7:2872. doi: 10.1038/s41598-017-02602-6 (PMC5460275; doi:10.1038/s41598-017-02602-6)

## Supplementary information - Nature Scientific Reports

# Genomic Selection in Commercial Perennial Crops: Applicability and Improvement in Oil Palm (*Elaeis guineensis* Jacq.)

Qi Bin Kwong[1*], Ai Ling Ong[1], Chee Keng Teh[1], Fook Tim Chew[2], Martti Tammi[1], Sean Mayes[3], Harikrishna Kulaveerasingam[1], Suat Hui Yeoh[4], Jennifer Ann Harikrishna[4,5*¶], David Ross Appleton[1¶]

[1] Biotechnology & Breeding Department, Sime Darby Plantation R&D Centre, Selangor, 43400, Malaysia

[2] Department of Biological Sciences, National University of Singapore, 117543, Singapore

[3] School of Biosciences, University of Nottingham, Sutton Bonington Campus, Loughborough, LE12 5RD, UK

[4] Institute of Biological Sciences, University Malaya, 50603, Kuala Lumpur, Malaysia

[5] Centre of Research in Biotechnology for Agriculture (CEBAR), University Malaya, 50603, Kuala Lumpur, Malaysia

* Correspondence: Qi Bin Kwong (kwong.qi.bin.@simedarby.com)

Jennifer Ann Harikrishna (jennihari@um.edu.my)

**Supplementary Table 1.** Selection gain estimated from all traits in study with selection threshold set as 25%.

| | M/F | *S/F | K/F | F/B | O/M | O/B | O/P |
|---|---|---|---|---|---|---|---|
| Selection response (%) | 4.95% | -17.91% | 24.79% | 4.49% | 4.72% | 6.68% | 14.65% |

***** Oil palm breeding aims to reduce S/F, and increase other yield traits.

**Supplementary Table 2.** Marker reduction by association for 4 traits.

*(Refer to separate spreadsheet – Supplementary Table 2.xlsx)*

**Supplementary Table 3.** Markers filtered by LD and the corresponding prediction accuracy for ascending number of markers for the traits of M/F and O/P.

| Number of markers | O/P without LD filter | O/P with LD filter | M/F without LD filter | M/F with LD Filter |
|---|---|---|---|---|
| 10 | 0.1684 | 0.2278 | 0.2965 | 0.3033 |
| 20 | 0.2700 | 0.2988 | 0.2789 | 0.3801 |
| 30 | 0.3270 | 0.3218 | 0.2800 | 0.4205 |
| 40 | 0.3321 | 0.3573 | 0.2740 | 0.4336 |
| 50 | 0.3426 | 0.3804 | 0.3078 | 0.4743 |
| 60 | 0.3883 | 0.3862 | 0.3887 | 0.5006 |
| 70 | 0.3970 | 0.3950 | 0.4045 | 0.5291 |
| 80 | 0.4218 | 0.4033 | 0.4109 | 0.5425 |
| 90 | 0.4255 | 0.4053 | 0.4473 | 0.5562 |
| 100 | 0.4260 | 0.4156 | 0.4494 | 0.5785 |
| 110 | 0.4280 | 0.4283 | 0.4545 | 0.5908 |
| 120 | 0.4344 | 0.4434 | 0.4443 | 0.5987 |
| 130 | 0.4403 | 0.4432 | 0.5386 | 0.6087 |
| 140 | 0.4426 | 0.4457 | 0.5594 | 0.6065 |
| 150 | 0.4483 | 0.4427 | 0.5709 | 0.6109 |
| 160 | 0.4571 | 0.4451 | 0.5705 | 0.6175 |
| 170 | 0.4674 | 0.4432 | 0.5721 | 0.6247 |
| 180 | 0.4761 | 0.4433 | 0.5736 | 0.6277 |
| 190 | 0.4821 | 0.4418 | 0.5755 | 0.6338 |
| 200 | 0.4813 | 0.4496 | 0.6250 | 0.6321 |
| 210 | 0.4774 | 0.4522 | 0.6439 | 0.6353 |
| 220 | 0.4794 | 0.4582 | 0.6521 | 0.6355 |
| 230 | 0.4768 | 0.4583 | 0.6432 | 0.6384 |

| | | | | |
|---|---|---|---|---|
| 240 | 0.4534 | 0.4567 | 0.6465 | 0.6419 |
| 250 | 0.4560 | 0.4596 | 0.6446 | 0.6419 |
| 260 | 0.4619 | 0.4625 | 0.6521 | 0.6435 |
| 270 | 0.4673 | 0.4656 | 0.6523 | 0.6423 |
| 280 | 0.4662 | 0.4688 | 0.6560 | 0.6461 |
| 290 | 0.4666 | 0.4689 | 0.6586 | 0.6449 |
| 300 | 0.4655 | 0.4699 | 0.6645 | 0.6480 |

**Supplementary Figure 1.** The distribution of kinship coefficients of 1,218 *tenera* palms.

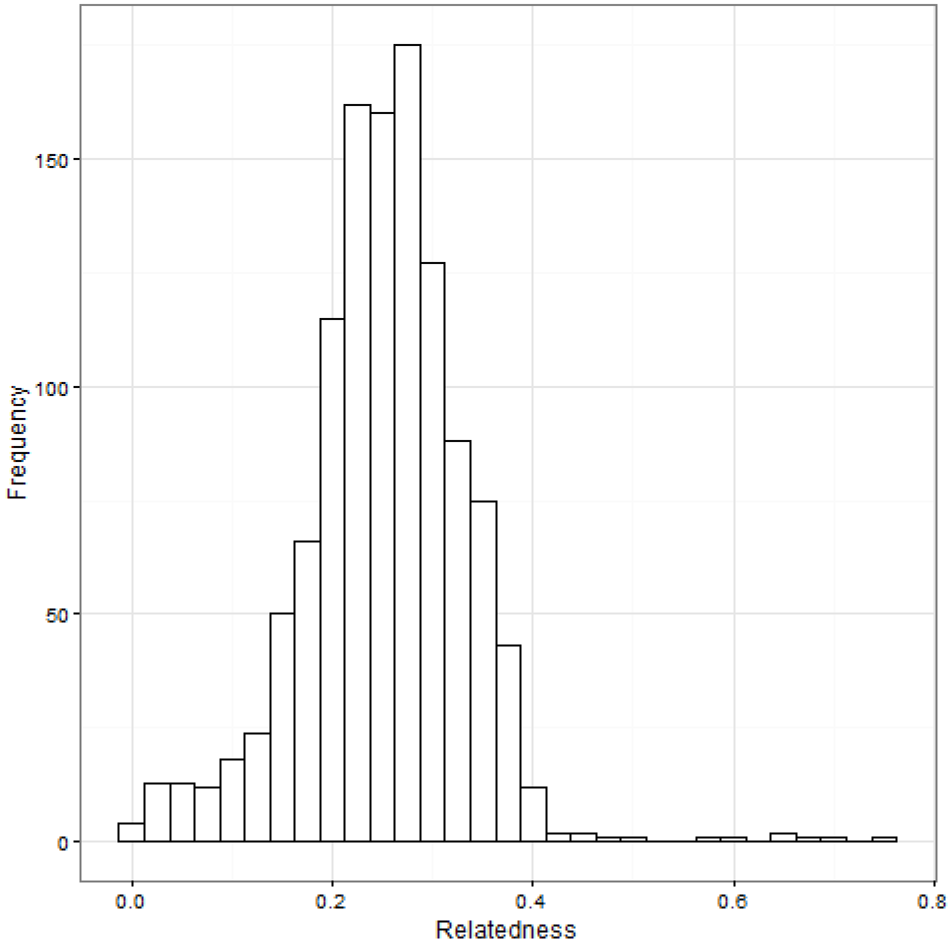

**Supplementary Figure 2.** Correlation between genomic heritability calculated from full marker set against marker subset.

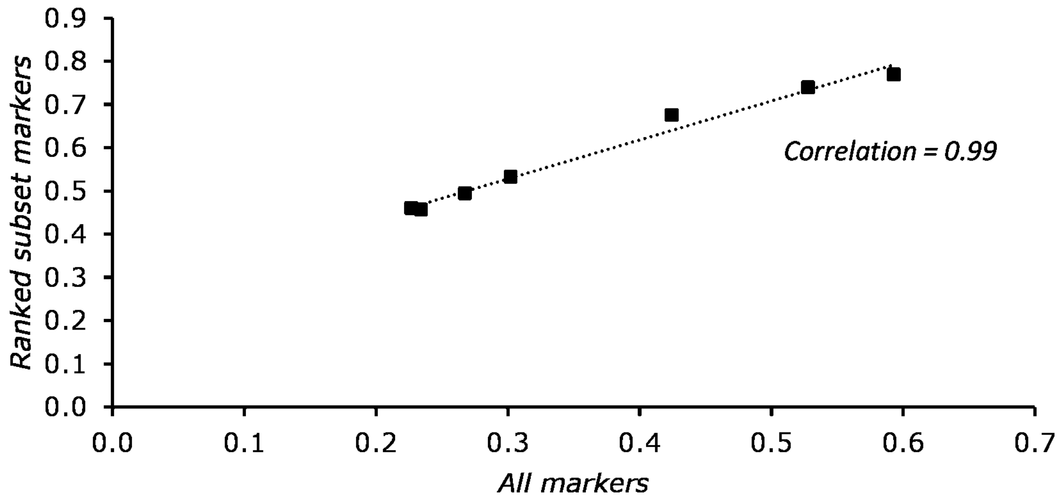

Supplement: Supplementary file 2 — Supplementary information [file 41598_2017_2602_MOESM2_ESM.pdf]
